# A fast, independent dose check of HDR plans

**DOI:** 10.1120/jacmp.v4i2.2530

**Published:** 2003-03-01

**Authors:** Martin E. Lachaine, Jason C. Gorman, Madeline G. Palisca

**Affiliations:** ^1^ Department of Radiation Oncology The University of Arizona Tucson Arizona 85724; ^2^ Department of Radiology University Medical Center Tucson Arizona 85724; ^3^ Department of Radiation Oncology The University of Arizona Tucson Arizona 85724

**Keywords:** HDR, brachytherapy, independent check

## Abstract

High dose rate (HDR) brachytherapy often involves optimization routines to calculate the dwell times and positions of a radioactive source along specified applicator paths. These routines optimize the dwells in such a way as to deliver the prescribed dose at one or more points while satisfying various constraints. The importance of independently verifying the doses calculated by the optimization software prior to treatment delivery has been recognized in various works, and is a requirement of various regulatory agencies. Most previous methods are specific to particular treatment configurations, or require a full replanning of the case. In this work we describe an in‐house software which provides an independent verification of dose calculations in less than 3 min, which adds negligible additional waiting time for the patient, regardless of the number of applicators, paths of the applicators, or complexity of the dwell times and positions. In order to verify errors which may occur between the planning and delivery stages, the verification code directly uses the treatment file used to control the HDR afterloader to compute the dose. Since this file references the source positions in the frame of reference of the catheters, an algorithm is described to convert these positions to Cartesian coordinates. We validate the code for various arbitrary cases ranging from a single catheter to complex multicatheter plans, and show results for various clinical plans. The maximum discrepancy observed for these clinical plans is 2%.

PACS number(s): 87.53.–j, 87.90.+y

## INTRODUCTION

High dose rate (HDR) brachytherapy treatment planning entails the identification of applicators on co‐registered simulator films or 3D images such as CT. The dwell times along these catheters are then optimized in an attempt to deliver prescribed doses at one or more anatomical points while simultaneously satisfying various constraints. The importance of independently verifying the dosimetry prior to treatment delivery has been recognized in various works, and is a requirement of various regulatory agencies such as the United States National Regulatory Commission (NRC).

There are two basic approaches to verifying HDR plans. The first relies on calculating a dose index or other characteristic parameter based on the variables defining the implant, such as total dwell time and activity.^1^ The calculated value for a given plan can then be compared to an expected value, depending on the type of implant. This expected value can be extracted from institutional experience for the given implant type. The second verification approach uses an independent dose calculation scheme to verify the dose at one or more points. The two verification approaches are complementary, since they both check different aspects of a plan; the first gives the user confidence that the plan is sensible, whereas the second makes sure that the dose is correct at critical points. It can be argued that the accuracy of dose calculations is verified upon commissioning of the treatment planning computer and checked at regular intervals. Checking the dose calculation on a per‐plan basis, however, ensures that the correct source is being used, that the source data has not been modified, that the correct activity, treatment date and decay are used, and that any bugs in the planning software did not affect the dose calculation.

In this work we focus on the second verification approach, i.e., verification of dose calculations prior to treatment. Most previously published techniques are specific to particular treatments, such as single catheters,^2^ ^–^ ^4^ GYN three‐catheter HDR,^5^ planar implants,^6^ or esophageal HDR.^7^ Saw *et al.* ^8^ published a technique which uses an LDR brachytherapy treatment planning system to re‐plan the patient using the optimized dwell times. Although this is an important general method to verify dosimetry, the length of time required to input the data for simple treatments is on the order of 20 min, which may be considered overly time‐consuming for routine use. Furthermore, the time required for replanning would increase dramatically for complex treatments with a large number of applicators, such as prostate HDR. Another issue which these published verification techniques do not address is the verification of the *treatment file* (the computer file generated by the treatment planning software to control the HDR afterloader). There is a possibility that either an error occurs in the generation of the file or that the wrong file is used.

In this article we describe an in‐house code used for the sole purpose of verifying HDR dosimetry. The code imports the positions of applicators and the optimized dwell times along these catheters, and calculates the dose at any desired point. The time required for verification is approximately 1–3 min, regardless of the number of applicators. This verification method adds negligible additional waiting time for the patient while providing a valuable independent check. In addition, the code uses the treatment file directly to obtain the dwell times and source positions to calculate the dose. An algorithm is described in the next section to convert these dwell positions, which are referenced along the paths of the applicators, to Cartesian coordinates to allow dose calculation.

## METHODS

### A. Description of verification code

In our clinic we use the VariSource HDR unit (Varian Medical Systems) along with BrachyVision (Varian Medical Systems) for treatment planning. For this reason, the in‐house verification code was designed specifically for the VariSource/BrachyVision combination, although it could easily be modified for other HDR units and/or planning software.

The BrachyVision planning system provides many different possibilities for dose optimization. Depending on the type of implant, we either (i) optimize using equal dwell times along applicators, (ii) use geometric optimization, which adjusts dwell times in an attempt to produce a uniform dose around applicators, or (iii) optimize by constraints, which attempts to deliver a specified dose to a point, a line, or multiple points and lines. We find it is often beneficial to alter the optimized plan by either “pulling” the isodose lines with the mouse, or by directly modifying the dwell times. The complex nature of this treatment planning process means that many approaches can be used to arrive at a final plan, making it possible that a bug not discovered during commissioning can affect the dosimetry if the planning steps are completed in a different order than usual. This makes a second check of the dose especially useful.

In order to verify errors which may occur between the planning and delivery stages, the in‐house verification code uses the treatment file (i.e., the file used to control the HDR afterloader during treatment) to directly compute the dose. Since this file references the source positions in the frame of reference of the applicators, but contains no information regarding the applicator paths, the code must convert the source locations to Cartesian coordinates. This requires extra information from the treatment planning computer regarding the paths of the applicators. The input to the verification code is thus (1) the source strength, reference date, and treatment date, which are entered independently by the user; (2) the treatment file; (3) text files containing the digitized points corresponding to the path of each applicator; and (4) coordinates of dose points.

The verification code uses the TG‐43 protocol^9^ for the calculation of dose rate, i.e.,

(1) $$\dot{D}(r,\theta )=S_{k}\Lambda \left[\frac{G(r,\theta )}{G(r_{0},\theta_{0})}\right]g(r)F(r,\theta ),$$

where $S_{k}$ is the air kerma strength of the source, Λ is the dose rate constant, *G*(*r, θ*) is the geometry factor, *g*(*r*) is the radial dose function, *F*(*r, θ*) is the anisotropy function, *r* is the distance between the dose point and the center of the source, *θ* is the angle subtended by the central axis of the source and the line connecting the center of the source and the dose point, and $r_{0}$ and $\theta_{0}$ are reference parameters (taken to be 1 cm and 90°, respectively). The air kerma strength radial dose function, and anisotropy function can be found in the literature for various commercial HDR sources. For example, our clinic uses the VariSource HDR system and we use the data of Wang and Sloboda^10^ for the 10 mm source and Angelopoulos *et al.* ^11^ for the 5 mm source. For the geometry factor, we use the equation for a linear source published by King, Anderson, and Mills,^12^ i.e.,

(2) $$G(r,\theta )=\frac{\sin^{-1}\left(\frac{L\cdot \sin\{\tan^{-1}[(r\cdot \sin\theta )/(r\cdot \cos\theta -L/2)]\}}{\sqrt{{\left[r\cdot \sin\theta \right]}^{2}}+{\left[r\cdot \cos\theta +L/2\right]}^{2}}\right)}{L\cdot r\cdot \sin\theta },$$

where *L* is the length of the source. Unfortunately, this solution diverges at θ=0 and thus, for this case we take the limit of Eq. (2) as *θ* tends to zero, i.e.,

(3) $$G(r,0)=\underset{\theta \to 0}{\lim}G(r,\theta )=\frac{1}{\left(r+L/2)(r-L/2\right)}.$$

Note that for the case r≫L this solution is in agreement with the geometry factor for a point source.

During the treatment planning stage, the locations of the applicators are digitized from co‐registered simulator films or from 3D images such as CT. The digitized points for the *i*th applicator can be represented as $\left(x_{\text{ij}}^{\text{dig}},y_{\text{ij}}^{\text{dig}},z_{\text{ij}}^{\text{dig}}\right)$, where the index *i* runs between 1 and $N_{\text{ap}}$, and the index *j* runs between 1 and $M_{i}$, where $N_{\text{ap}}$ and $M_{i}$ are the number of applicators and the number of digitized positions along the *i*th applicator, respectively. Once the points are digitized into the treatment planning computer, an optimized plan is generated. A treatment file, which is used to control the afterloader during treatment, is then written to a diskette. This file contains the required dwell positions $l_{\text{ik}}^{\text{dwell}}$ and associated dwell times $\Delta t_{\text{ik}}$ along the paths of the applicators, referenced from the distal end of the *i*th applicator. Here the label *i* refers once again to the applicator number and the label *k* indexes the dwell positions along the path of that applicator. The label *k* runs from 1 to the number of dwell positions along the *i*th applicator, $N_{i}$.

Assuming these digitized points $\left(x_{\text{ij}}^{\text{dig}},y_{\text{ij}}^{\text{dig}},z_{\text{ij}}^{\text{dig}}\right)$ are connected by straight lines, one can transform the dwell positions $l_{\text{ik}}^{\text{dwell}}$ into Cartesian coordinates $\left(x_{\text{ik}}^{\text{dwellg}},y_{\text{ik}}^{\text{dwell}},z_{\text{ik}}^{\text{dwell}}\right)$ by first finding the indices of the digitized points j=J and j=(J+1) which bound the given dwell position. This is accomplished by finding the distances of these points along the applicator, $l_{\text{ij}}^{\text{dig}}$, which satisfy

(4) $$l_{J}^{dig}⩽I_{ik}^{dwell}<I_{i,(J+1)}^{dig},$$

where

(5) $$l_{iJ}^{dig}=\sum_{J=1}^{J-1}\sqrt{{\left(x_{ij}^{dig}-x_{i,(j+1)}^{dig}\right)}^{2}+{\left(y_{ij}^{dig}-y_{i,(j+1)}^{dig}\right)}^{2}+{\left(z_{ij}^{dig}-z_{i,(j+1)}^{dig}\right)}^{2}}+l_{tip}.$$

The length $l_{\text{tip}}$ represents the distance between the end of the applicator and the most distal source position.

**Figure 1:**
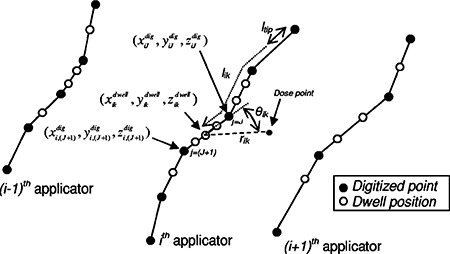
Schematic diagram of geometry used for dose algorithm.

Once the indices of the two bounding points *J* and (J+1) are found, the $\left(x_{\text{ik}}^{\text{dwell}},y_{\text{ik}}^{\text{dwell}},z_{\text{ik}}^{\text{dwell}}\right)$ can be calculated by interpolating along the line segment joining these two points (see Fig. 1), i.e.,

(6) $$x_{ik}^{dwell}=x_{iJ}^{dig}+\left(\frac{x_{i,(J+1)}^{dig}-x_{iJ}^{dig}}{l_{i,(J+1)}^{dig}-l_{iJ}^{dig}}\right)(l_{ik}^{dwell}-l_{iJ}^{dig})$$

and similarly for $y_{\text{ik}}^{\text{dwell}}$ and $z_{\text{ik}}^{\text{dwell}}$. With these Cartesian dwell coordinates $\left(x_{\text{ik}}^{\text{dwell}},y_{\text{ik}}^{\text{dwell}},z_{\text{ik}}^{\text{dwell}}\right)$ and corresponding dwell times $\Delta t_{\text{ik}}$, the dose at any point (*x,y,z*) can be calculated using

(7) $$D(x,y,z)=\sum_{i=1}^{N_{ap}}\sum_{k=1}^{N_{i}}\dot{D}(r_{ik},\theta_{ik})\Delta t_{ik},$$

where $\overset{\cdot }{D}(r,\theta )$ is calculated from Eq. (1), $r_{\text{ik}}$ and $\theta_{\text{ik}}$ are the distance and angle between the source location and the dose point respectively (see Fig. 1), i.e.,

(8) $$r_{ik}=\sqrt{{\left(x-x_{ik}^{dwell}\right)}^{2}+{\left(y-y_{ik}^{dwell}\right)}^{2}+{\left(z-z_{ik}^{dwell}\right)}^{2}}$$

and

(9) $$\cos\theta_{ik}=\frac{r_{1}\cdot r_{2}}{\left\|r_{1}\right\|\;\left\|r_{2}\right\|},$$

where

(10) $$r_{1}\equiv (x_{iJ}^{dig}-x_{ik}^{dwell},y_{iJ}^{dig}-y_{ik}^{dwell},z_{iJ}^{dig}-z_{ik}^{dwell})$$

and

(11) $$r_{2}\equiv (x-x_{ik}^{dwell},y-y_{ik}^{dwell},z-z_{ik}^{dwell}).$$

**Figure 2:**
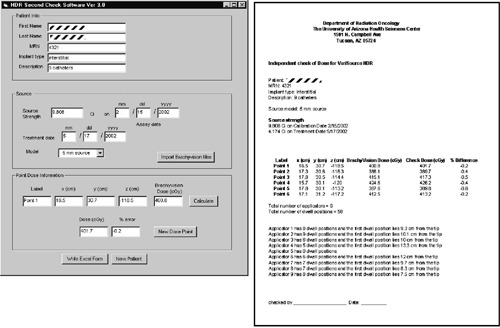
Left, Screenshot of HDR check software; right, print‐out generated by HDR check software.

The graphical user interface (GUI) windows‐based software [Fig. 2(a)], written using Visual Basic (Microsoft Corp), requests the activity and date information, reads the text files, and calculates the dose at any point. It subsequently generates a form [Fig. 2(b)] with the planned dose, verification dose, and associated percent error for the points of interest, which is used as a formal verification of the treatment dosimetry. The time required for verification is on the order of 1 min regardless of the complexity of the plan or the number of applicators.

### B. Code evaluation

To evaluate the check software, we first compared its calculations to a single dwell position calculated using the BrachyVision planning system. We then used it to validate the calculations for four plans with arbitrarily complex geometries. The plans were much more complex (many catheters with tortuous paths and sharp discontinuous turns) than would be used clinically in order to push the limits of the calculation code. Each plan had up to seven dose verification points.

To demonstrate the utility of the software for clinical cases, we use it to verify the dose for three vaginal cylinder cases, one endometrial case, one endobronchial case, one intraluminal case (all based on orthogonal simulator films), and two CT‐based nine‐catheter interstitial implants. Depending on the complexity of a given plan, we have chosen between two and five points per plan to verify the dose.

## RESULTS

For single‐dwell position plans, the in‐house verification software agrees with the BrachyVision calculations within 1%, except for points lying along the source axis (θ=0), where discrepancies on the order of 10% are observed. This discrepancy occurs because the anisotropy function at θ=0 is not given in the tables used and so the value is very sensitive to the extrapolation method. When the anisotropy on both the planning and check algorithms are turned off, this discrepancy disappears.

**Figure 3:**
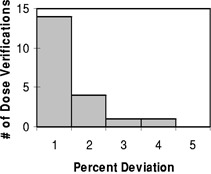
Histogram representing the percent deviation of the dose calculated by the verification code compared to the planned dose for a total of 20 dose points from the complex nonclinical plans.

The percent deviation between the dose calculated by BrachyVision and that calculated by the verification software for the complex nonclinical plans are shown in Fig. 3. It is seen that all dose points are within 4%, and that 14 out of 20 fall within 1%. This is good agreement considering the tortuous paths chosen for the applicators in these plans.

Good agreement was found for the clinical plans. For the plans based on orthogonal films, all of the dose points were well within 1% of the planned dose. For the two CT‐based implants all dose points were also within 1% of the planned dose except for two dose points, which were within 2%.

## DISCUSSION

The code described in this article is a quick, useful method to check the dose calculation prior to each treatment. It requires approximately 1–3 minutes, and thus does not significantly increase the patient's waiting time. It must be stressed, however, that the software check is only one part of a complete patient quality assurance protocol. For instance, the software uses the same coordinate system, digitized applicator paths, and dose point coordinates as the treatment planning system and thus will not pick up errors such as incorrect digitization of applicators, incorrect position of dose points, or improper magnification of simulation films. These types of errors can, however, be discovered by inspection of the treatment plan printouts and the simulation films.

The main utility of the second check philosophy implemented in this article is to gain confidence that the dose calculation is accurate. Even though the accuracy of dose calculations is fully verified upon commissioning and during periodic quality assurance tests, checking the dosimetry prior to each treatment assures that (1) the correct source is being used; (2) the source data has not been modified; (3) the correct activity, treatment date and decay are used, since these data are entered independently in the check program (this is important with BrachyVision since it keeps track of the activity decay automatically); (4) that no errors occur in the creation of the treatment file since the software uses this file directly to calculate the dose; (5) that the treatment file corresponds to the correct plan; and (6) that any bugs (known or unknown) in the planning software did not affect the dose calculation. In addition, some extra features have been built in to the code to assist in the treatment QA: the number of applicators, the number of dwell positions for each applicator, and the distance of the first dwell positions for each applicator, referenced from the distal end, are printed out which can be used to verify against the treatment plan [see Fig. 2(b)].

## CONCLUSION

A dedicated in‐house software is described which independently checks the dosimetry at select points for HDR plans. The code uses the optimized dwell times and positions along the applicators directly from the treatment file used to control the afterloader during treatment. The code is written to work with a specific treatment planning system, but can conceivably be modified for other systems.

Other than some discrepancies along the source axis, the source modeled the point source within 1% of the treatment planning computer. The code was also tested for various nonclinical plans designed to test the limits of the algorithm. These plans had multiple catheters and complex applicator paths. Most dose points (14 out of 20) were within 1%, with a maximum discrepancy of 4%. The code was used to verify eight clinical plans, using between 1 and 8 applicators, with a maximum discrepancy of 2%. This shows that the code is a quick way of independently checking dosimetry, which is an important part of a complete quality assurance program for HDR brachytherapy.
